# Hospital-Based Ultra-Sonographic Prevalence and Spectrum of Thyroid Incidentalomas in Pakistani Population

**DOI:** 10.7759/cureus.17087

**Published:** 2021-08-11

**Authors:** Faheemullah Khan, Kiran Hilal, Iftikhar Ali, Mehreen Samad, Rabiya Tariq, Wiqar Ahmad, Muhammad Arif Saeed, Noman Khan

**Affiliations:** 1 Radiology, Aga Khan University Hospital, Karachi, PAK; 2 Pharmacy Unit, Paraplegic Center, Peshawar, PAK; 3 Radiology, Hayatabad Medical Complex Peshawar, Peshawar, PAK; 4 Internal Medicine, Lady Reading Hospital, Peshawar, PAK; 5 Radiology, James Paget University Hospitals, NHS Foundation Trust, Norfolk, GBR

**Keywords:** thyroid, nodule, ultrasound, ti-rads, incidentalomas

## Abstract

Introduction: Thyroid incidentalomas (TIs) are clinically asymptomatic nodules found accidentally during imaging studies ordered for some other reasons. Being easily accessible, non-invasive, and inexpensive, thyroid ultrasound (US) is a key investigation in the management of thyroid nodules.

Methods: This ultrasound-based cross-sectional study was performed in the radiology department of a major tertiary care hospital. Every second patient visiting the emergency department was a potential candidate for a thyroid ultrasound. Patients having ages greater than 20 years were included in the study.

Results: A total of 250 patients were included in the study. Out of these, 175 were female and 75 were male. The majority (54.80%) were in the age group 21-30 years. Nodules were found in 65 (26%) patients and in the majority of cases (67.7%) they were multiple in number. Associated lymphadenopathy was seen in only one patient. Thyroid nodules were more common in females as compared to males (75.38% versus 24.62%). According to Thyroid Imaging and Reporting Data System (TI-RADS) classification, the majority of the nodules were falling in TI-RADS 1 (74%) followed by TI-RADS 3 (9.60%) and 4A (8.80%).

Conclusion: The thyroid nodules are more commonly seen in females as compared to males. A significant association is seen between the frequency of thyroid nodules and increasing age. The majority of thyroid nodules fall in TI-RADS 1 category followed by TI-RADS 3 and 4A.

## Introduction

Thyroid incidentalomas (TIs) are clinically asymptomatic nodules found accidentally during imaging studies ordered for some other reasons [1,2]. Thyroid nodules, according to the American Thyroid Association (ATA) are “discrete lesions within the thyroid gland, radiologically distinct from surrounding thyroid parenchyma” [3]. Being easily accessible, non-invasive, and inexpensive, ultrasound (US) is an essential investigation in the evaluation of thyroid nodules (TNs). Ultrasound is important for not only the detection but also for the stratification of these nodules [1,2]. The nodules may vary a lot according to morphology, physiology, and histology. TN may be single or multiple, solid or cystic, and benign or malignant. TN may be physiologically functioning or non-functional depending on whether it is secreting thyroid hormone or not [4,5]. Ultrasound examination is limited, that is, it can reliably comment only on the number and morphology of TNs. However, applying the established reporting system called TI-RADS (Thyroid Imaging and Reporting Data System) can give the probability of a nodule being likely benign or malignant [6,7].

The prevalence of TNs vary greatly, largely due to the different methods of identification. Until the 1980s, palpation alone was used for TN detection. With the introduction of ultrasonography, we saw an apparent epidemic of nodules. Although approximately 4% to 7% of the general population has a palpable TN, this occurrence increases to almost 76% with the use of ultrasonography [5,7,8]. TNs are now increasingly identified with the increase in the use of imaging studies for the evaluation of neck pathologies.

Thyroid nodules are clinically important for several reasons, however, their primary importance is due to their risk of malignancy. The reported prevalence of malignancy in TNs evaluated by biopsy ranges from 4.0% to 6.5% and is largely independent of the nodule size [9,10]. In Pakistan, there is scarcity of data regarding the burden of TIs and its risk stratification. To the best of our knowledge, there is only one well-documented study on the prevalence of TIs conducted in Karachi, Pakistan [4]. However, they neither utilize the TI-RADS nor did they advocate their utility. The aim of this study is to know the burden of TIs in the adult Pakistani population and describe their sonographic spectrum based on TI-RADS.

## Materials and methods

This ultrasound-based cross-sectional study was conducted in Hayatabad Medical Complex (HMC), from April to May 2019. Hayatabad Medical Complex is located in Peshawar, the capital of Khyber-Pakhtunkhwa province of Pakistan. It is one of the three major tertiary care public sector hospitals of Khyber-Pakhtunkhwa province. HMC is a 1280-bedded hospital that receives patients from all over Khyber-Pakhtunkhwa as well as from neighbouring Afghanistan. Patients visiting the emergency ultrasound room of Hayatabad Medical Complex for scans other than thyroid scans were provided with information and informed consent regarding the said study. The thyroid ultrasound was done free of charge. Every second patient visiting the emergency department was a potential candidate (probability sampling). Patients having ages greater than 20 years were included in the study. Those having palpable thyroid nodules, thyroid malignancy and diffuse thyroid disease or any history of these were excluded. Verbal informed consent was a must for the patients.

Ethical approval

The study protocol was approved by the ethical review committee of Hayatabad Medical Complex (Reference Number: 627, Dated February 02, 2019). The study was protecting the anonymity of the participants.

Procedure and technique

The ultrasound was performed with linear probes of 3-12 MHz. The machine that was used for the thyroid ultrasound was Mindray-DC-70 (Mindray Bio-Medical Electronics Co., Ltd., Shenzhen, China). Images obtained were saved for review later on. Every scan was interpreted initially by the operator who was a resident well trained on the thyroid ultrasound to meet the study requirements and then reviewed later by the same resident and two other experienced radiologists (one having five and the other at least three years experience). The participants were examined in a supine position with their neck hyperextended both in axial and longitudinal view. Length, width, and height of the thyroid lobes along with isthmus thickness were noted. TNs were documented according to TI-RADS. The routine reporting of thyroid nodules in the department is not according to the TI-RADS criteria. A data collection form consisting of 10 items was developed with the help of published literature on the same topic. The said document was filled out for each participant. The items/variables were: age, gender, district, thyroid dimensions (length/width/height), nodules (present or absent, single or multiple), ultrasound characteristics of nodules, adenopathy, vascularity, and TI-RADS classification for each TI (the higher TI-RADS category in case of multiple nodules).

Statistical analysis

Data analysis was done with SPSS®V 20.0 (IBM Corp., Armonk, New York). Mean ± SD, numbers, and proportions were calculated through descriptive statistics. The chi-square test was applied to assess the association of thyroid nodules with age and gender. The comparison of prevalence of two gender groups was performed using the Fisher exact test. Results were documented in numbers and percentages for categorical variables. Differences were considered statistically significant if p < 0.05.

## Results

**Figure 1:**
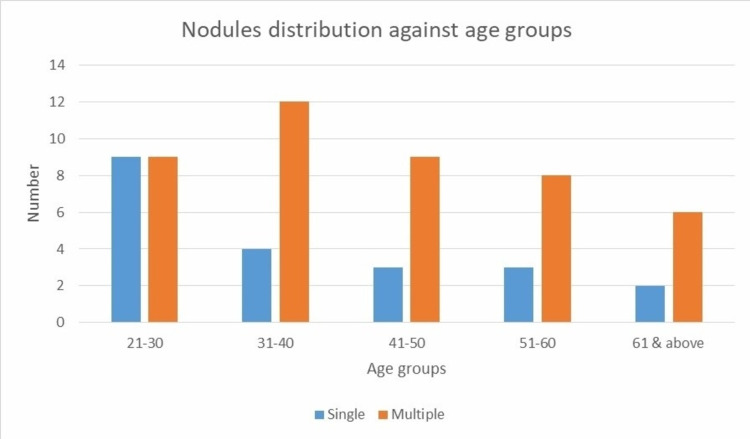
Single versus multiple nodules.

A total of 250 patients were included in the study. Out of these 175 were female and 75 were male. Excluding six Afghani patients, all were Pakistani citizen. Majority of them (95.2%) were of Phatan ethnicity. Majority (54.80%) were in the age group (21-30 years). Mean width of the right thyroid lobe was 17.84 ± 4.52 (SD) and that of the left lobe was 16.59 ± 4.24 (SD). The mean width of the thyroid was 15.38 ± 3.41 (SD). The mean thickness of the isthmus was 2.99 ± 1.17 (SD). Nodules were found in 65 (26%) patients. In majority of them having nodules in their thyroid gland, nodules were multiple in number (67.7%; Table 1 and Figure 1).

**Table 1:** Description of nodules.

Variable		Number	Percentage
Nodules	Present	65	26%
	Absent	185	74%
Number of nodules	Single	21	32.2%
	Multiple	44	67.7%
Nodule characteristic	Solid	33	50.77%
	Cystic	4	6.15%
	Mixed	28	43.08%
Adenopathy	Present	1	1.5%
	Absent	64	98.5%
TI-RADS	1	185	74%
	2	15	6%
	3	24	9.60%
	4A	22	8.80%
	4B	3	1.20%
	5	1	0.40%

The TI-RADS distribution of the nodules is given in Table 1. Associated lymphadenopathy was seen in only one patient. Frequency of thyroid nodules was higher in older age groups (p˂0.001; Table 2). Thyroid nodules were more common in females as compared to males (75.38% versus 24.62%).

**Table 2:** Group wise distribution of thyroid nodules.

Age group (years)	Nodules		Total	p-value
	Present	Absent		
20-30	18 (13.1%)	119 (86.9%)	137	˂0.001
31-40	16 (29.1%)	39 (70.9%)	55	
41-50	12 (44.4%)	15 (55.6%)	27	
51-60	11 (68.8%)	15 (31.3%)	16	
61 and above	8 (53.3%)	7 (46.7%)	15	

## Discussion

With the advent of US, the detection of thyroid nodules has increased. Being non-invasive and inexpensive, the US has an important role in the assessment of thyroid nodules, that can even detect nodules as small as 2-3 mm. Ultrasound examination of thyroid for nodules assessment includes; presence or absence of internal calcification, echogenicity, vascularity, and associated lymphadenopathy. The introduction of linear probes for thyroid ultrasound examination has remarkably improved the visualization of pathology and its details [5].

The prevalence of thyroid nodules varies, depending on sex, age, and demographics. We found thyroid nodules in 26% of the participating patients. Previously Kamran et al. had reported a 21% prevalence of thyroid nodules in a hospital-based population on ultrasound examination [4]. Studies from other regions (Iran, USA, Korea, Poland, and Finland) have reported prevalence of thyroid nodules as 13.6%, 9.4%, 36.67%, 14.8%, and 27.35%, respectively [11-15]. We found that the frequency of thyroid nodules increases with increasing age (p˂0.001), a finding that has been reported by many across the globe [4,11,14,16]. Thyroid nodules were seen more commonly in female participants as compared to males. This has been observed by many assessing thyroid nodules prevalence [4,11,14,16]. The gender difference has been explained to be related to both estrogen and progesterone. The evidence provided is the increasing nodule size and new nodule development during pregnancy and with multiparity [5].

We observed that in the case of thyroid nodules, the majority were having multiple thyroid nodules, a finding that is in contrast to what has been observed previously by many authors [4,11,12,15]. This finding may be related to the difference in demographics or the reason might be that the hospital-based cohort is not representative of the original population.

According to the TI-RADS classification proposed by Horvath et al. [6], the majority of the nodules were falling in TI-RADS 1 (74%) followed by TI-RADS 3 (9.60%) and 4A (8.80%). The application of TI-RADS is important to stratify and streamline patients, whether they require further workup or no workup. Patients in the category of TI-RADS 3 were advised follow-up with their primary physician and those of higher categories were advised fine needle aspiration cytology as further workup after prior consultation with their primary team. The major public health challenge regarding thyroid nodules is to highlight the importance of further workup in case of nodules with suspicious features on ultrasound. The poor literacy rate and socioeconomic status of our population make it hard for public health professionals to ask people to seek to check-up for a problem that looks benign, i.e., asymptomatic thyroid nodule. The second big challenge is for the hospitals and the radiological society to encourage the reporting of thyroid nodules as TI-RADS. Here, reporting of single or multiple thyroid nodules which is the norm, adds little value to the report. If TI-RADS reporting can be performed on ultrasound in an emergency setting as in our case, why that cannot be done in an outpatient setting. Further studies targeting the wider out of hospital population are required.

Limitations

It is worth mentioning that a limitation of the current pilot study is that the research was carried out on patients who presented to the emergency department and were advised ultrasound examination other than thyroid, so the results of this study may not be representative of the general population. Although the primary reason to perform this study was to know the burden of disease, i.e., thyroid nodules, which is the first step toward solving a health problem, we could not perform the biopsies for thyroid nodules with higher TI-RADS category as the patient's primary purpose for presenting in the emergency room was not related to thyroid. The protocol was used for research purposes and highlighting the importance and incorporation of TI-RADS into the radiological reports.

## Conclusions

The thyroid nodules were more commonly seen in females as compared to males. A significant association was seen between the frequency of thyroid nodules and increasing age. The majority of thyroid nodules were in TI-RADS 1 followed by TI-RADS 3 and 4A. The study highlights the great burden of non-palpable and clinically asymptomatic thyroid nodules. It is high time that TIRADS reporting is introduced across the country.
